# Associations between commonly consumed beverages and the oral microbiome in three US cohort studies

**DOI:** 10.64898/2026.10.07.26364990

**Published:** 2026-10-09

**Authors:** Lynn E. Ferro, Samuel Anyaso-Samuel, Yukiko Yano, Xing Hua, Yunhu Wan, Vicky C. Chang, Casey L. Dagnall, Kristine Jones, Belynda D. Hicks, Amy Hutchinson, Linda M. Liao, Wen-Yi Huang, Neal D. Freedman, Laura E. Beane Freeman, Dale P. Sandler, Christian C. Abnet, Jianxin Shi, Rashmi Sinha, Erikka Loftfield, Emily Vogtmann

## Abstract

Multiple external factors shape human microbial communities. Beverages represent particularly frequent dietary exposures that may impact oral microbiome composition. We examined associations of coffee, tea, alcohol, sugar-sweetened beverages, artificially-sweetened beverages, and fruit juice consumption with the oral microbiome in three large cohort studies (n=5,598). Beverage consumption was assessed by food frequency questionnaire and categorized as non-consumers and tertiles of intake among consumers, except fruit juice, which was categorized into quartiles. DNA from oral wash specimens was sequenced (16S rRNA, V4 region). Associations with alpha and beta diversity and individual genera were assessed using multivariable linear, logistic, count-based regression models and weighted permutation-based models accounting for multiple comparisons in genus-level analyses. Only alcoholic beverage consumption was associated with all alpha diversity metrics; the highest tertile of alcohol drinking was associated with 10.18 (95% CI: 5.56, 14.80) greater observed amplicon sequence variants vs. non-consumers. All beverages accounted for 4.06% of variability in beta diversity (unweighted UniFrac); in univariable models, alcoholic beverages had the largest R² among individual beverages (0.86%) and accounted for the majority (25/28, 89.3%) of genus-level associations, mostly with beer and liquor. For example, high alcoholic beverage intake (T3 vs. non-consumers) was associated with 2.25 and 2.17 times the odds of having *Megasphaera* and *Peptostreptococcus* , respectively, and 23% lower abundance of *Lautropia* . Alcoholic beverage consumption was strongly associated with oral microbes, including taxa linked to poor oral health; these findings suggest that alcohol-associated differences in the oral microbiome may have implications for oral and systemic health.

## Full Text Availability

The license terms selected by the author(s) for this preprint version do not permit archiving in PMC. The full text is available from the preprint server.

